# The Intestinal Dysbiosis of Mothers with Gestational Diabetes Mellitus (GDM) and Its Impact on the Gut Microbiota of Their Newborns

**DOI:** 10.1155/2021/3044534

**Published:** 2021-09-22

**Authors:** Xinke Li, Da Yu, Yushuang Wang, Huimin Yuan, Xixi Ning, Binqi Rui, Zengjie Lei, Jieli Yuan, Jingyu Yan, Ming Li

**Affiliations:** ^1^Dalian Medical University, Dalian, Liaoning 116044, China; ^2^Department of Obstetrics, Dalian Women and Children's Medical Group, Dalian, China; ^3^Department of Obstetrics and Gynecology, Suihua First Hospital, Suihua, China; ^4^Dalian Institute of Chemical Physics, Chinese Academy of Sciences, Key Laboratory of Separation Science for Analytical Chemistry, Dalian, China

## Abstract

Gestational diabetes mellitus (GDM) is defined as “diagnosed as impaired glucose tolerance for the first time during pregnancy,” which can lead to adverse pregnancy outcomes and produces divergent effects on mothers and newborns. In recent years, with the continuous expansion of obese people, GDM shows an upward trend. The abundant and diverse members of the human gut microbiota exert critical roles in the maintenance of human health. Studies have shown that GDM may be associated with disordered gut microbiota in both mothers and newborns. Taking into account the potential effects on maternal and consequently neonatal health, in this review, we analyzed the available data and discussed the current knowledge about the potential relationship between GDM and intestinal dysbiosis in mothers and newborns. In addition, we also discussed the influencing factors derived from GDM mothers on the gut microbiome of their newborns, including the vertical transmission of microbiota from mothers, the alteration of milk components of GDM mothers, and using of probiotics. Hoping that new insights into the role of the gut microbiota in GDM could lead to the development of integrated strategies to prevent and treat these metabolic disorders.

## 1. Introduction

Gestational diabetes mellitus (GDM) is an increasing public health concern that affects approximately 5∼20% of pregnancies [1]. The prevalence of GDM has continued to increase during the past few decades and is likely to see a further rise in the future. GDM affects both mother and child with short-term complications such as preeclampsia, cesarean delivery, neonatal hypoglycemia, and congenital malformation, while long-term complications included maternal T2DM and cardiovascular diseases, as well as obesity, and other metabolic diseases in the offspring [2]. Metabolic disturbance usually occurs in GDM women, including decreased insulin secretion and increased insulin resistance, which are typically related to obesity/overweight [3].

There are rich and diverse microbiota in the gut of humans, which play an important role in maintaining human health. A substantial body of evidence supports that gut microbiota plays a pivotal role in the regulation of metabolic, endocrine, and immune functions. The gut microbiota can use polysaccharides in food, and they produce short-chain fatty acids (SFCAs) by fermenting and absorbing polysaccharides. Studies in mice have shown that SFCA supplementation improves insulin sensitivity and dyslipidemia, prevents weight gain, and increases energy expenditure in diet-induced obese mice [4]. Depletion of SCFA-producing bacterial species might therefore contribute to the increased inflammatory tone often found in patients with obesity and diabetes. Changing the quantity and quality of the gut microbiota destroys the homeostasis of the gut environment and leads to the occurrence or development of many human diseases. In recent years, people have become increasingly aware of the importance of the microbiota during pregnancy and early life, as they are closely related to reproductive health. The early colonization of the microbiota may affect the development of newborns and may cause long-term adverse consequences in the future [5]. At present, most studies have analyzed the effects and related mechanisms of GDM on mothers, but few studies on infants (especially the effect on the gut microbiota of infants). The present review analyzes the correlation between changes of the gut microbiota in mothers with GDM and their infants. In particular, we focus on the possible influencing aspects of GDM mothers on the gut microbiota in infants, including the vertical transmission of maternal microbiota, breastfeeding, and the use of probiotics. We aim to prompt the development of innovative therapeutic targets for the slowing of adverse effects of GDM by highlighting the role of the gut microbiota in GDM infants.

## 2. The Influencing Factors and Adverse Pregnancy Outcome of GDM

The well-documented risk factors for GDM include prepregnancy body mass index (BMI) within the range of overweight or obesity, advanced maternal age, family history of diabetes, or any form of diabetes and cigarette smoking [6]. Genetic factors are also one of the risk factors of GDM. At present, we have found some genes related to GDM, but they are very limited [7]. We are aware of only one published genome-wide association study (GWAS) of GDM to date. This was conducted among Korean women and demonstrated a potentially shared genetic basis between GDM and type 2 diabetes [8].

GDM is related to diverse adverse pregnancy outcomes for both the mother and their kids. For the mother, gestational diabetes increases the risk of obstetrical complications such as preterm delivery and dystocia. For infants, fetal macrosomia is a common adverse infant outcome in GDM, which is more likely to be large and macrosomic, and infants more easily suffer from shoulder dystocia, clavicle fracture, and brachial plexus injury at birth [9]. After birth, infants from GDM mothers are likely to develop childhood obesity, metabolic syndrome, T2DM, and impaired insulin secretion [9]. Emerging, yet suggestive data indicate that these children may be at high risk for atopic dermatitis and allergen sensitization. The clinical study found that GDM infants are more sensitive to allergens and their sensitization risk increases more than 5-fold. It is also more likely to suffer from atopic dermatitis, which increases its risk by more than 7-fold [10].

## 3. The Gut Microbiota of Pregnant Women with GDM

### 3.1. Changes of Gut Microbiota in Normal Pregnant Women

During pregnancy, the body of pregnant women undergoes weight and metabolism changes, which is accompanied by changes in the gut microbiota. Weight gain during pregnancy was positively correlated with the relative abundance of *Bacteroides*, *Escherichia coli*, and *Enterobacteriaceae* [11] and negatively correlated with the abundance of *Bifidobacterium* and *Akkermansia muciniphila* [12]. The gut microbes in early pregnancy are similar to those in nonpregnant women; however, O. Koren et al. found that intestinal microbiota changed in the early and third trimesters of pregnancy, characterized by increased diversity (*β* diversity) and decreased richness (*α*-diversity) in pregnant women [13]. Some obesity-related bacteria such as Actinobacteria and Proteobacteria Phyla were found to increase significantly in the third trimester of pregnancy. Notably, the researchers also reported a decrease in butyric acid-producing bacteria *Faecalibacterium* that have an anti-inflammatory activity in pregnant women [14]. All of these changes seem to lead to weight gain (high obesity) and insulin resistance (IR) in pregnant women, which mainly occur in the third trimester of pregnancy.

Confirming the influence of microbiota on metabolic function, Koren et al. transplanted fecal samples from early and late pregnant women into germ-free mice and found that mice with late gestational fecal samples were more likely to be obese and more likely to induce inflammation [13]. At present, the relationship between different metabolic variables of pregnancy and some specific bacteria has been found; for example, there is a negative correlation between insulin values and *Blautia*; arterial blood pressure and *Odoribacter*; and ghrelin insulin and *Prevotellaceae.* One study conducted a prospective observational and exploratory study of 41 patients with GDM and found that there was a correlation between C-reactive protein and *Sutterella*; circulating levels of insulin and *Collinsella*; and ghrelin and *Bacteroidaceae* [15]. Therefore, gut microbiota may affect the changes of some metabolic indexes during pregnancy in different ways, but the internal mechanism is still unclear and needs further study.

### 3.2. Changes of Gut Microbiota in Pregnant Women with GDM

Some metabolic changes in pregnancy promote the accumulation of adipose tissue in the early stage. With the advancing of gestational age, the ability to decompose fat in the body increases. In the third trimester of pregnancy, the ability of insulin to prevent fat decomposition is inhibited, which is further aggravated in women with GDM, resulting in an increase in free fatty acids in the body of pregnant women, accelerating the production of hepatic glucose and severe insulin resistance (IR). This severe IR has been found to be associated with a decline in the numbers of *Roseburia* and *Faecalibacterium prausnitzii* in the third trimester of pregnancy in women with GDM, which are butyric acid-producing bacteria with anti-inflammatory properties [16]. In addition, studies have reported that chronic low-grade inflammation in women with gestational diabetes mediates an imbalance in tryptophan metabolism. The study found that the maternal tryptophan–kynurenine pathway was upregulated in women diagnosed with GDM compared with the control group [17]. Some specific bacteria have the ability to produce tryptophan, such as *Escherichia coli*. In the intestinal tract, it has also been clearly proved that major tryptophan metabolism pathways such as 5-hydroxytryptamine and kynurenine are directly or indirectly regulated by microbiota [18]. It is suggested that the imbalance of the array of metabolites in GDM is closely related to the microbiota.

The changes of the gut microbiota in women with GDM compared with those without GDM have been reported.  Table 1 shows the specific changes in intestinal microbes in GDM mothers shown in these studies. Compared with healthy pregnant women, the gut microbial community diversity of women with GDM changed, including the decrease of *α*-diversity and the increase of *β*-diversity. In addition, GDM also showed various types of abnormal bacterial composition, including changes in phylum, genus, and species levels. At the phylum level, an increase in Firmicutes/Bacteroidetes (F/B) ratio in late pregnancy was exhibited in the GDM group when compared with non-GDM [19]. As reported, a higher F/B ratio is more likely to cause obesity and aggravate inflammation. At the genus level, some intestinal bacteria such as *Parabacteroides*, *Prevotella, Haemophilus,* and *Desulfovibrio* are more abundant in women with GDM when compared with those of healthy women in both second and third trimesters of pregnancy [16, 19–22]. Most of these are Gram-negative bacteria. Lipopolysaccharide (LPS) is a unique structure exposed to the outer membrane of the cell wall of Gram-negative bacteria and is an important endotoxin of most intestinal pathogens. It is highly immunogenic and stimulates B lymphocytes to produce specific antibodies, resulting in low-grade inflammation and IR. LPS has strong immunogenicity and can stimulate B lymphocytes to produce specific antibodies, which can contribute to low-grade inflammation and insulin resistance [23]. At the individual level, the biosynthesis and transport system of LPS have always been positively correlated with the blood glucose level of the oral glucose tolerance test (OGTT) [21]. Meanwhile, the relative abundance of SCFA-producing genera such as *Faecalibacterium*, *Ruminococcus*, *Roseburia*, *Coprococcus*, *Akkermansia*, *Phascolarctobacterium*, and *Eubacterium* in the gut of GDM women was significantly lower than that of the healthy women [2, 16, 19–21, 24, 25]. These changes are reported to be associated with elevated blood glucose levels in individuals [16, 19–21].

Therefore, changes in the intestinal microbiota during the first trimester of pregnancy may be considered as a potential diagnostic tool for GDM or may be one of the causes of GDM. However, previous studies have shown that the gut microbiota composition of women diagnosed with GDM at early pregnancy is similar when compared with those of women without GDM at the same gestational stage [26], suggesting that the imbalance of the gut microbiota may be a result of GDM. Therefore, the debate about whether intestinal microbiota is the cause or consequence of gestational diabetes is still unclear, and further research on their relationship is needed.

## 4. The Gut Microbiota of GDM Infants

### 4.1. Early Colonization of Gut Microbiota in Healthy Infants

The early colonization of intestinal bacteria in infants usually occurs at birth. In the first few days, only a few groups of alien microbes, unrelated to the source of nutrition, settled in the intestines and became more stable in the first week of life. At that time, facultative anaerobes belonging to *Enterobacteriaceae*, *Streptococcus*, *Staphylococcus*, and *Enterococcus* already existed, mainly due to the initial supply of oxygen in the gut of newborns [27]. *Escherichia coli*, *Enterococcus faecium,* and *Enterococcus faecalis* are the most represented species in the first batch of colonizers.

With the gradual increase of oxygen consumption of facultative anaerobes, there is an anoxic environment in the gut, which leads to an increase in some obligate anaerobes such as *Bifidobacterium*, *Bacteroides,* and *Clostridium* [27]. With the introduction of solid food, the colonization and diversity of bacteria in the gut have undergone continuous changes, and one of the most prominent features is the increase in the number of *Bacteroides*.

Among the gut bacteria in the early stage of healthy infants, *Bifidobacterium* is the dominant bacteria in the colonization microbiome. *Bifidobacterium* appeared on the 3rd-4th day after birth, then increased gradually, and peaked in the first year. With the increase of age, the number of *Bifidobacterium* began to decrease in the second year, other intestinal microbiota species began to expand, and the intestinal microbial community of infants became more diversified [28].

*Bifidobacteria* and *Lactobacilli* contribute to both natural and acquired immune responses in healthy neonates. According to the research report, there is an association between a low level of fecal *Bifidobacteria* in the early stage and a high risk of noncommunicable diseases (such as atopic diseases and obesity) in the later stage [29]. The presence of *Bifidobacteria* in the human adult intestinal microbiota is minor, indicating that *Bifidobacteria* is specific for early life [30].

### 4.2. Changes of Gut Microbiota in GDM Infants

A large number of convincing experimental data show that maternal metabolic disorders are closely related to the development of related metabolic diseases such as obesity in offspring [31]. Some metabolic diseases that mothers often suffer from, such as gestational diabetes, overweight, or obesity, increase the offspring's risk of metabolic disorders associated with inflammation and weight gain. The establishment of intestinal barrier function and the maturation of the immune system depend on early bacterial colonization [32]. Early colonization is the decisive factor of mucosal dynamic balance. So it is particularly important to observe the effect of GDM on gut microbiota in infants.

Some previous studies have reported significant changes in intestinal microorganisms in the offspring of mothers with GDM, including a decrease in *α*-diversity and changes in the relative abundance of some specific bacteria.  Table 2 shows the specific changes in intestinal microbes in the offspring of mothers with GDM shown in these studies.

Ponzo et al. [33] have found that GDM infants showed a higher relative abundance of proinflammatory bacterial taxa and a lower *α*-diversity than infants from healthy women. Hu et al. [34] collected the first intestinal discharge from 23 newborns stratified by maternal diabetes status and found that the maternal diabetes status was significantly associated with the relative abundance of *Bacteroidetes*. Wang et al. [5] found an increase in the number of lactic acid bacteria in the meconium of newborns of mothers with GDM, indicating that some specific colonizing bacteria in the gut of infants may be affected by maternal GDM status. Su et al. [35] have found that there are differences in gut microbiota between the newborns from GDM mothers and the control group. The gut microbiota of the GDM infants showed lower *α*-diversity than that of the control group. At the phyla level, the abundance of Proteobacteria and Actinobacteria increased and that of Bacteroidetes decreased in the GDM group. Besides, a few unique gut microbiota belonging to the phylum of Proteobacteria, Firmicutes, and Actinobacteria were found in the neonatal fecal samples of healthy infants and were absent in GDM ones. At the genus level, the number of *Prevotella* and *Lactobacillus* decreased in newborns from GDM mothers.

Correlation analysis showed that maternal fasting blood glucose levels had a positive correlation with the relative abundance of phylum Actinobacteria and genus *Acinetobacter* but a negative correlation with the relative abundance of *Bacteroidetes* and genus *Prevotella*. Finally, the study also found that the total amount of bacteria in newborns differs significantly from the severity of diabetes in mothers [35]. Intestinal dysregulation not only leads to various gastrointestinal diseases, such as acute diarrhea and chronic enteritis, but also leads to the occurrence of several metabolic syndromes and neurogenic diseases, including obesity, hyperglycemia, and autism [36]. Previous studies have reported changes in the gut microbiota in children with diabetes and found a significant decrease in the number of *Lactobacillus* and *Prevotella* [37]. In addition, another study reported that gastrointestinal diseases caused by autism may be related to the absence of *Prevotella* [38]. These results are consistent with the changes in the gut microbiota in infants with GDM. It is speculated that the variation of these intestinal bacterial genera may be related to diabetes and gastrointestinal diseases, which may lead to a higher risk of these diseases in neonates with GDM than in control newborns. The results of these studies are of great significance for understanding the internal relationship of GDM with neonatal gut microbiota and thus on their future healthy development, which is worthy of in-depth study.

## 5. The Maternal Factors That Affect Gut Microbiota of GDM Infants

**Figure 1:**
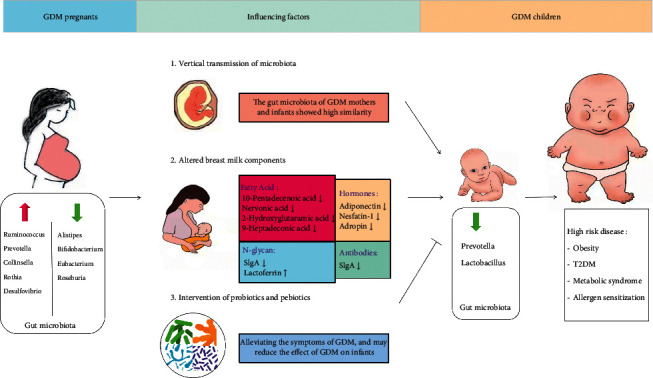
GDM mothers influence the gut microbiota of infants in different ways.

It is well known that the maternal internal environment affects the health of offspring. The intestinal microbiota of newborns is strongly affected by maternal health and pregnancy status and participates in the developmental programming of the newborns. Overweight, obesity, and allergies in children are related to maternal/newborn dysbiosis. Many prenatal and postnatal factors have been shown to affect the colonization of early intestinal microbiota in infants, such as mode of delivery and breastfeeding. GDM is the most common complication of pregnancy, which increases the risk of metabolic disorders such as obesity and diabetes in offspring. At present, there is little data on the relationship between maternal characteristics of GDM and neonatal microbiota. Next, we will break down the following points to introduce the effect of GDM mothers on infants' gut microbiota in different ways, as shown in Figure 1.

### 5.1. Vertical Transmission of Maternal Microbiota

The early colonized microbiota is important for the establishment and maturation of metabolic pathways. Evidence from analysis of experimental data supports that the vertical transmission of microbiota from mother to offspring is an important source of early colonization of infant gut microbiota [36]. In this context, Azad et al. collected fecal samples from 24 Canadian healthy infants at 4 months of age and found that some microbes including *Bifidobacteria, Clostridium,* and viral organisms have a different genetic diversity between mothers and infants of different individuals but have the same genetic characteristics in mothers and their babies [39]. Some animal experiments found that, compared with the control group, lower levels of *Lactobacillus* appear in the vagina of maternal mice which are exposed to stress, and the abundance of *Lactobacillus* in the gut of the mother was positively correlated with the abundance of *Lactobacillus* in the gut of offspring [33].

In another mouse study [40], the researchers fed pregnant mice normal milk or milk containing genetically tagged bacteria, then obtained the fetuses by aseptic cesarean section, and analyzed their fecal samples. The results showed that fecal samples from mothers fed milk containing genetically tagged bacteria were found to contain the same genetically tagged bacteria, which was not detected in the control mothers or children. However, despite the consensus view of vertical transmission from mother to infant, knowing the exact source of early colonizers and the modes of transmission is still a challenge. Given the potential relationship between the vertical transmission of the maternal microbiome and the gut microbiota of infants, more research on the mechanism is needed.

The gut microbiota, which is the most abundant of microbial flora in the body, may be a potential source of the transmission of mother-to-infant bacteria [41]. The gut has barrier properties, which can prevent harmful substances passing through the intestinal epithelium. During pregnancy, the intestinal permeability of mothers increases, which leads to an increase in the ability of intestinal contents to cross the intestinal epithelial barrier. The placental endothelial integrity also changes during pregnancy, which may allow the bacteria from the gut to cross the barrier into umbilical cord blood and amniotic fluid [42]. Therefore, it was speculated that maternal bacteria may participate in vertical transmission by crossing the placenta [43]. It has been found that antibiotic resistance genes in maternal intestinal bacteria can be detected in fecal samples of newborns. Through the study of mother-infant pairs, Ferretti et al. longitudinally sampled the microbiome of 25 mother-infant pairs across multiple body sites from birth up to 4 months postpartum in the Italy cohort and found that on the day of delivery, the proportion of the gut microbial species of the infant that were transmitted from the mother reached up to 50.7%, and this fraction was relatively stable over the next 4 months [44]. The most contribution was from the mothers' gut, accounting for 22.1% [44]. Roswall et al. studied with a larger population size of 98 mother-infant pairs and reported that 72% of the colonized microbial species in the vaginally delivered infant gut within 2–5 days after birth were shared species, such as *Bifidobacterium longum, Bacteroides fragilis*, and *Enterococcus faecalis* [45]. The number of other species that are not transmitted from mother to baby is very low and drops to an undetectable level after four months [45]. In addition, six species consisting of three *Bacteroides species (B. uniformis, B. vulgatus, and B. dorei*), two *Bifidobacterium species (B. adolescentis* and *B. longum*), and *E. coli* were found in pairs of mothers and infants in the Finnish cohort [46]. Microbiota from the maternal gut are more persistent over time compared to other maternal sources [45]. Although unrelated individuals often shared the same species, the number of species shared by infants and their mothers in the first three days was significantly higher than that shared with other mothers [44].

Vertical transmission of maternal microbes has been confirmed to be widespread, so it is worth exploring whether vertical transmission of maternal microbes will have an impact on newborns of GDM mothers. The clinical study of the intestinal microbiota of mothers and offspring of GDM recently revealed that there were two shared genera including *Bacteroides* and *Brucellosis* colonized in GDM mothers and their babies, suggesting that GDM offspring have maternal microbial imprints. The study also showed that abundant proinflammatory microbial groups appear in the gut of infants with GDM, such as *Escherichia coli* and *Parabacteroides,* compared with the infants of the healthy mother [33]. Another study [5] investigated the possibility of maternal and neonatal microbiota disorders associated with GDM by collecting and analyzing samples from 581 pregnant women (oral, intestinal, and vaginal) and 248 newborns (oral, pharynx, meconium, and amniotic fluid) and estimated the potential risk of microbial transfer to newborns. The study revealed that there were a large number of high abundant OTUs that vary with the same trend by counting maternal and neonatal microbiota, in which *Prevotella*, *Streptococcus*, and *Bacteroides* are the most common genus in the tested samples, reflecting the consistency of microbiological variation between mother and infant. In addition, the study calculated correlations between bacterial genera in samples from mothers and neonates with and without GDM. Notably, the proportion of the microbiota which had the same cooccurrence trend between generations reached up to 88.8%, in which 69.1% were only detected in GDM+ but not in GDM−. Despite body part-specific variations, the effects of GDM on maternal and neonatal microbiota may be similar. Animal experiments also confirm the above point of view. Yao et al. established a GDM mouse model and explored the effect of GDM on the gut microbiota of maternal mice [47]. It was found that the *Bacteroides* and *Clostridiales_vadinBB60* were more abundant, while *Prevotella* was much lower in GDM mice than in control mice. However, most of these bacteria were found to have a common trend in GDM offspring; it was found that *Bacteroides* were more abundant, while *Lactobacillus* and *Prevotella* were less abundant in the gut of offspring fed by GDM mothers [48]. GDM can change the microbiota of pregnant women and newborns, revealing another mode of heredity. However, there are few studies on the vertical transmission of GDM maternal microbiome to newborns, and more data are needed to be analyzed.

### 5.2. Effect of Breast Milk of GDM Mothers on the Gut Microbiota of Their Offspring

Breast milk plays an important role in the growth and development of infants. In addition to providing the nutrients that babies need, breast milk also provides complex carbohydrates and proteins, which have a wide range of biological activities and can promote the development and maturity of the infant immune system, as well as early healthy intestinal colonization [49].

Breastfeeding may affect the composition of gut microbiota. One study found that *Bifidobacteria* and *Clostridium difficile* are more abundant in breastfed newborns, whereas *Bacteroides* and *Clostridium perfringens* prevail in formula-fed infants [50]. The difference of intestinal microbiota in infants with healthy mothers caused by different feeding methods also existed in infants with GDM. One study compared the gut microbiota of newborns of 29 GDM puerpera (10 were breastfed and 19 were formula-fed) and found that there were differences in gut microbiota between breastfed babies and formula-fed babies.

At the phylum level, breastfed infants showed more Actinobacteria and Proteobacteria, while in formula-fed infants, we observed a higher proportion of Firmicutes phyla. At the genus level, breastfed infants showed more *Escherichia* and *Bifidobacterium*, while formula-fed infants had different microbiota composed mainly of *Bacteroides*, *Clostridium*, *Enterococcaceae*, *Escherichia*, *Streptococcus*, *Staphylococcus*, and *Streptococcus*. In the multiple regression analysis, breastfeeding was significantly associated with the relative abundance of *Bifidobacterium* in the intestinal microbiota of the 11 infants (*P*=0.0017). Remarkably, the breastfed infants had a higher number of *Bifidobacterium* compared with the formula-fed infants, which was considered to have a positive effect on babies. However, in comparison with the infants of healthy women, a higher relative abundance of proinflammatory taxa was shown in infants with GDM, such as *Escherichia* and *Parabacteroides* [33]. This may be related to the change in the composition of breast milk. Next, we will analyze it from this perspective.

#### 5.2.1. Breast Milk Oligosaccharides and Glycans

Human Milk Oligosaccharides (HMOs) are free oligosaccharides with multiple biological functions, which are the third largest component of human milk. It is completely indigestible to newborns but can be used by some intestinal bacteria. In addition to HMOs, the glycoprotein is another large source of breast milk glycobiome. Glycoprotein is a kind of protein in which one or more sugars are connected to the peptide chain by a covalent bond. According to the connection mode, the glycans on glycoproteins are divided into N-polysaccharides and O-polysaccharides. It was found that more than 70% of human milk proteins are glycosylated, and human lactose proteins play a defensive role against infectious diseases by producing antibacterial and immunomodulatory activities of passive immunity to breastfed infants [48]. Breast milk glycobiome has been shown to selectively enrich the infant gut microbiome with beneficial bacteria [51]. These beneficial bacteria are able to quickly consume HMOs as the sole carbon source and successfully become the dominant bacteria in the gut [52]. However, some intestinal bacteria consume HMOs poorly or not at all, such as *Clostridium perfringens*, *E. faecalis,* and *Veillonella parvula* [52]. In this way, breast milk oligosaccharides help babies establish a healthy gut environment.

Due to the different abilities of intestinal microbiomes to use different types of glycans as carbon sources for growth and metabolism [48], differences in breast milk glycans may also affect the composition of intestinal microbial communities in offspring. In this context, some research groups have studied the glycobiome patterns in the breast milk of mothers with GDM, it was found that [53], compared to healthy women, the content of free oligosaccharides in the breast milk of GDM women was not different, but the total protein concentration and glycosylation level of sIgA in GDM breast milk were reduced; in contrast, the glycosylation of lactoferrin in the milk of GDM mothers was increased compared with the breast milk of healthy control mothers. They found that the content of total N-glycan of sIgA was 32–43% lower than that of normal pregnant women (*P* < 0.0001), and the content of total N-glycan of lactoferrin was 45% higher than that of normal pregnant women. These results suggested that maternal glucose regulation disorder has been happening in GDM women during pregnancy. Because breast milk glycobiome is closely related to the gut microbiota of infants, differences in milk glycans may also affect the composition of the gut microbiome in the offspring. However, there are few studies at present.

Previously, our team established a GDM mouse model and collected milk and fecal samples of GDM maternal and offspring mice to observe the changes of oligosaccharides and protein N-glycans in the milk of GDM mice and their possible effects on the gut microbiota of offspring [48]. Different from the main proportion of fucosylated milk oligosaccharides in human milk, mouse milk mostly contains sialylated milk oligosaccharides. We found that there are no significant differences in the abundance of milk oligosaccharides between the CON and GDM mice, which is consistent with the findings in human milk. However, through further analysis, we found the levels of fucosylation and sialylation of N-glycan in the milk of GDM mice were significantly higher than those of CON mice. On this basis, we analyzed the gut microbiota of offspring mice. Our results showed that the abundance of *Bacteroides spp*. was significantly increased in the gut of offspring mice fed by GDM mothers when compared with those fed by healthy control mothers. *Bifidobacteria* and *Lactobacillus* are the major microbial genera in the gut of healthy breastfed infants. They promote the healthy growth and development of babies, and the decrease of these bacteria may indicate the poor state of newborns. On the contrary, some *Bacteroides* spp. have the strong ability to use complex polysaccharides, which promotes their growth in the gastrointestinal tract. So large amounts of fucosylated and sialylated N-glycans may provide a major carbon source for *Bacteroides,* resulting in their dominance in the intestines of newborns fed GDM. In particular, through further experiments in vitro, we found that the metabolites of *Bacteroides* could stimulate the lymphocyte, whereas they inhibit the production of Treg cells. Treg cells can inhibit the immune response of other cells and maintain the immune balance of the body [54]. Our results suggest an immune imbalance in the offspring with GDM, which may be a predisposing factor for this type of disease. However, the mechanism of action is still not clear, which is worthy of our more in-depth study.

#### 5.2.2. Antibodies in Breast Milk

Newborns are exposed to an environment that contains a large number of viruses and bacteria when they are born. Lacking a mature immune system, newborns initially rely on antibodies transferred by their mothers. These antibodies are transmitted through the placenta and breast milk. In the placenta, the mother mainly transmits IgG, which helps to prevent neonatal infection [55, 56]. In addition, other studies have also shown that the mother transfers IgE to the fetus through the placenta, which is closely related to neonatal allergies [57]. After birth, the baby continues to gain maternal immunity through breast milk. Unlike placentally transferred IgG, BM mainly contains SIgA, which plays a leading role in neonatal mucosal immunity. The antibodies in breast milk populate the intestinal mucosal surface of newborns, providing the first line of defense for the healthy development of the intestinal system in the early stages of infants.

The antibody concentration in breast milk changes dynamically throughout the lactation period according to the needs of the baby. In colostrum, the antibody content is high, while in mature milk, the antibody concentration of breast milk decreases, replaced by an increase in carbohydrates and fat. Antibodies in breast milk are mainly synthesized by plasma cells in the breast. Recently, increasing evidence shows that a large part of antibodies in breast milk are related to antigen specificity of intestinal origin [58]. The mother selectively transfers mucosal immunity-related antibodies to the baby through breast milk, which provide a barrier against the same antigens found in the mother's environment, which newborns are most likely to encounter.

Increasing evidence shows that immunoglobulin plays a key role in the establishment and maintenance of early healthy microbiota in infants. Maternal immunoglobulin selectively wraps microorganisms in the small intestine, promotes the colonization of symbiotic bacteria, and delivers antigens to antigen-presenting cells, thus inhibiting the proliferation of pathogens. Most of the SIgA in the mucosa is considered to be nonspecific, highly cross-reactive, and widely reactive with the microbiota. Through a process called immune exclusion, SIgA captures microbes and enables the immune system to selectively sample complex bacteria to produce immunity by limiting the translocation of bacteria between mucosal epithelial cells [59]. In addition, SIgA can cause immune rejection to viruses and bacteria by promoting pathogens to gather or neutralize pathogens in the intestinal lumen [60]. Unlike IgA, IgG promotes tolerization by forming IgG-allergen complexes promoting the uptake of allergens by epithelial cells and assisting in the immune presentation of allergen [61].

In the individuals with IgA deficiency, *Enterobacter* accounted for a higher proportion of the microbiota, which is the dominant bacteria in the infant's gut [62]. Interestingly, an increase in the incidence of allergies and autoimmune diseases has been observed in patients with IgA deficiency, which may be the result of this change in microbiota [63]. Similar results have occurred in the infant from GDM women, which may be closely related to the change of antibody concentration in the breast milk of GDM mothers. It was found that the level of SIgA in the breast milk of GDM patients was significantly lower than that of healthy controls. Therefore, antibodies in breast milk are essential to promote the development and maintenance of healthy intestinal microbiota in infants [53].

#### 5.2.3. Free Fatty Acids in Breast Milk

Free fatty acids are the main nutrients in breast milk, which are very important for the growth and development of newborns. Some studies have shown that free fatty acids in breast milk may affect early intestinal microbiota colonization in infants [64]. In one study, Heerup et al. examined the effect of selected nonesterified fatty acids, monoacylglycerols, and sphingosine on the composition of fecal microbial communities derived from infants aged 2–5 months during a 24 h anaerobic in vitro fermentation.

The results showed that the number of acid-producing bacteria such as *Lactobacillus* and *Bifidobacterium* increased significantly in the presence of a high concentration of medium-chain nonesterified fatty acids. In the mixture containing long-chain nonesterified fatty acids and sphingosine, *Bifidobacterium* was also found to increase significantly. However, the relative abundance of *Enterobacteriaceae* decreased significantly in the presence of the mixture of two lipids. It is also worth noting that oleic acid (18 : 1), the most common fatty acid in human milk, has been found to stimulate the growth of several types of *Lactobacillus* [65]. These findings suggest that the high concentration of nonesterified fatty acids in breast milk might have functional effects on the establishment of the gut microbiota in early life. In the early stages of life, the establishment of the immune system is very important for growth and development. It may be very beneficial to promote the growth of lactic acid-producing bacteria such as *Bifidobacterium* and *Lactobacillus* and reduce the number of *Proteobacteria* in the intestinal microbiota. One study [66] compared the metabolites of colostrum, transitional milk, and mature milk between normal pregnant women (*n* = 94) and GDM women (*n* = 90). The results showed that quite a lot of free fatty acids in breast milk significantly declined in the GDM group compared to the control group. It is suggested that there is a disorder of fatty acids in the breast milk of mothers with GDM. However, the effect of disturbed fatty acids in GDM breast milk on gut microbiota in infants has not been reported and needs to be further explored.

#### 5.2.4. Hormones in Breast Milk

Hormones in breast milk were suggested to protect infants from the short-term acceleration of adipose deposits and long-term obesity and diabetes. Some studies have assessed hormone levels in breast milk in women with GDM. Adiponectin and ghrelin concentrations were found to decrease in the breast milk of pregnant women with GDM [67]. And adiponectin was inversely associated with early infant growth in both women with GDM and healthy babies who grow up with low levels of adiponectin in the breast milk of GDM women and are more likely to be obese than healthy babies. However, with favorable controlled blood glucose, breastfeeding can help babies of women with GDM regain a healthy growth trajectory [68]. Aydin [69] evaluated the concentration of Nesfatin-1 in the breast milk of GDM rats, which is a peptide that derives from the precursor peptide nucleobindin 2. It has been found that Nesfatin-1 has an anorexia effect on rats and can make rats lose weight [70].

The authors found that the concentration of Nestitin-1 in the colostrum of rats with GDM was significantly lower than that of non-GDM rats, while the concentration of Nestitin-1 in the mature breast milk of GDM rats was lower, but the difference was not statistically significant, which might be due to the normalization of their blood glucose over time [69]. Thus, in the first week of life, offspring fed with breast milk with lower levels of Nesfatin-1 may be more likely to be hungry, so they drink more breast milk than those who are fed normal breast milk. Previous studies have shown that the concentration of plasma Nesfatin-1 in newborns is negatively correlated with the degree of hunger (calorie intake). Obese patients tend to have lower circulating Nesfatin-1 levels and higher calorie intake [71]. The same group of investigators evaluated adropin concentrations in the breast milk of GDM mothers. Adropin is a peptide hormone that is involved in the regulation of metabolic homeostasis [72].

Aydin et al. [73] found that the adrenaline concentration in the colostrum of GDM women was lower than that in non-GDM women, but the adropin level in immature milk during the transitional period (7 days after delivery) was not different between the two groups. Adrenaline deficiency has been shown to be associated with increased fat content in mice, suggesting that exposure to lower levels of adrenaline in GDM breast milk may also lead to the increased fat content in children [72]. These suggest that the level of breast milk hormone in parturient women with GDM is a disorder, which leads to an increase in the probability of obesity in infants. It is generally believed that obesity can cause disorders of the gut microbiota in infants; therefore, we speculate that the disorder of hormone levels in breast milk may affect the gut microbiota of infants. Luoto et al. reported differences in adiponectin concentrations in the maternal colostrum and in fecal *Bifidobacteria* counts at age 3 months between normal children (*n* = 15) and overweight children (*n* = 15) [74]. The authors found that higher *Bifidobacteria* was detected in normal children at the age of 3 months compared with overweight children, and the level of adiponectin in breast milk was significantly higher in mothers with normal children than in those with overweight children. These results suggest that hormones in breast milk have a more complex effect on the gut microbiota of an infant than previously anticipated. However, there is little research in this area, and more data is needed to support it.

### 5.3. Intervention of Probiotic and Prebiotics

At present, the microbial intervention has received widespread attention. Probiotics usually contain live, freeze-dried bacterial microbes, mainly from intestinal beneficial bacteria such as *Lactobacillus and Bifidobacterium*. When given sufficient amounts of probiotics, probiotics regulate and promote the intestinal health of the host. During pregnancy, most pregnant women use probiotics orally, and few use vaginal administration. Probiotic interventions are not live bacteria but are made up of indigestible food substances that can be broken down into HMOs and used by beneficial bacteria in the intestines, thereby promoting the expansion of these beneficial bacteria. Synbiotics combine probiotics and probiotics intervention. Synbiotics promote the survival of living microorganisms in the intestinal tract by stimulating the growth and/or metabolic activity of one or more probiotics, thus producing beneficial effects. Probiotics intervention measures were used during pregnancy, and probiotics were used to a lesser extent to improve maternal and infant outcomes.

Recently, using probiotics to prevent or treat GDM has become a hot research direction. Dolatkhah et al. enrolled 64 pregnant women with GDM into the clinical trial and randomly divided them into three groups, which were treated with probiotics capsule or placebo capsule and dietary advice for 8 weeks. They found that fasting blood glucose and insulin resistance index decreased significantly in patients treated with probiotic capsules or placebo capsules (*P* < 0.05) [75]. Luoto et al. also found that probiotic intervention reduced the risk of GDM [76]. Specific probiotic therapy may change the composition and activity of intestinal microbiota, to improve the intestinal microecological environment, repair the intestinal barrier, and enhance the intestinal ability to regulate inflammation. Recently, the gut microbiota are considered one of the keys to participate in the dynamic balance of host energy, affecting the acquisition of energy from the outside and storage in the body. In addition, it also regulates plasma endotoxin concentration and insulin sensitivity to prevent the occurrence of metabolic syndrome. Considering that the maternal microbiota is the first inoculum to the development of the child's microbiota, GDM mothers receiving probiotic intervention during pregnancy may promote the establishment of early healthy intestinal microbiota in infants. A systematic review and meta-analysis looking at the effect of treatment of GDM on pregnancy outcomes showed that treatment significantly reduced the risks of fetal macrosomia, large-for-gestational-age births, shoulder dystocia, and gestational hypertension, as well as a tendency to reduction of perinatal/neonatal mortality and birth trauma [77]. A review of probiotics for the prevention of GDM included one study that reported lower rates of women diagnosed with GDM and lower birth weight with probiotics [78]. These results suggest that probiotics taken by GDM mothers during pregnancy can reduce the adverse pregnancy outcome and promote the healthy growth of the baby. Taking probiotics may be a good way to prevent or treat GDM, and more research on the mechanism is needed.

## 6. Summary

Altered gut microbial structures of the GDM mother and their offspring have been proved by many studies, and some of the changed bacteria have the same trend in the intestines of the mother and their offspring, which suggests that the mother's microbiome may be transmitted to the child, reflecting the influence of the GDM mother's gut microbiota on the colonization process of the child's gut microbiota. This provides a new direction for early prevention and treatment of GDM to reduce the incidence of adverse pregnancy outcomes in GDM. At the same time, we found that changes in the composition of GDM breast milk have a potential impact on the healthy development of babies, which may provide a theoretical basis for future studies aimed at developing specific nutritional care for children of mothers with gestational diabetes.

## Figures and Tables

**Table 1:** Comparison of changes in the gut microbiota between GDM women and non-GDM women.

Surveyed country	No.		GW (weeks)		Features of gut microbial community		Reference
	G+	G−	G+	G−	Increase	Decrease	
China	11	11	31.2 ± 0.5	32.7 ± 0.3	Verrucomicrobia (P) *Akkermansia* (G)	*Faecalibacterium* (G)	[20]

China	43	81	26.2 ± 1.2	25.9 ± 1.9	*Parabacteroides* (G) *Megamonas* (G) *Phascolarctobacterium* (G) *Streptococcus agalactiae* (S) *Lachnospiraceae bacterium* (S)	*Ruminiclostridium* (G) *Roseburia* (G) *Fusobacterium* (G) *Haemophilus* (G) *Clostridium* (G) *Bifidobacterium* (S) *Eubacterium siraeum* (S) *Alistipes shahii* (S)	[21]

Denmark	50	157	28.7 ± 1.4	28.4 ± 1.1	Actinobacteria (P) *Collinsella* (G) *Desulfovibrio* (G) *Blautia* (G) *Ruminococcus* (G)	*Bacteroides* (G) *Faecalibacterium* (G) *Ruminococcus* (G) *Isobaculum* (G)	[16]

Brazil	26	42	32.45 ± 7.04	28.23 ± 5.68	Firmicutes (P) *Ruminococcus* (G) *Collinsella* (G) *Lachnospiraceae* (G) *Dorea* (G)	Bacteroides (P) *Eubacterium rectale* (G)	[19]

China	74	73			*Fusobacterium* (G) *Prevotella* (G)	*Faecalibacterium* (G)	[5]

China	23	26	38.6–39.7	39.0–40.6	*Bacteroides dorei* (S)	*Alistipes putredinis* (S) *Lactobacillus casei* (S)	[25]

China	30	31	38.3 ± 0.7	38.5 ± 0.8	*Haemophilus* (G)	*Alistipes* (G) *Rikenellaceae* (G)	[22]

China	36	16	25.6 ± 1.0	25.9 ± 1.1	*Blautia* (G)	*Faecalibacterium* (G) *Phascolarctobacterium Roseburia* (G)	[2]

China	45	45	25.55 ± 1.17	25.68 ± 1.26	*Blautia* (G) *Faecalibacterium* (G)	Bacteroides (P) *Akkermansia* (G) *Odoribacter* (G) *Butyricimonas* (G)	[26]

China	31	103	24.5 ± 0.5	24.5 ± 0.5	*Holdemania* (G) *Megasphaera* (G) *Eggerthella* (G)	*Streptococcus* (G)	[24]

No., number; G+, GDM; G−, non-GDM; GW, gestational weeks; P, phylum; G, genus; S, species. The increased/decreased microbiota in GDM women when compared with non-GDM.

**Table 2:** Changes of gut microbiota in the newborns of GDM mothers compared with the newborns of mothers without GDM.

Surveyed country	No.		Features of gut microbial community		Ref.
	G+	G−	Increase	Decrease	
China	24	24	*Lactobacillus iners* (S)		[5]

Italy	29	19	Actinobacteria (p) Bacteroidetes (p) *Escherichia* (G) *Parabacteroides* (G)	*Staphylococcus* (G) *Ralstonia* (G) *Lactobacillus* (G) *Enterobacteriaceae* (G)	[33]

America	5	13	Bacteroidetes (p)		[34]

China	20	14	Actinobacteria (p) Proteobacteria (p)	Bacteroidetes (p) *Prevotella* (G) *Lactobacillus* (G)	[35]

No., number; G+, GDM; G−, non-GDM; P, phylum; G, genus; S, species. The increased/decreased microbiota in the newborns of GDM mothers when compared to the newborns of mothers without GDM.
